# Controllable Nitrogen-Doped Hollow Carbon Nano-Cage Structures as Supercapacitor Electrode Materials

**DOI:** 10.3390/molecules30102130

**Published:** 2025-05-12

**Authors:** Yitong Sun, Xiaoqin Niu, Laidong Yang, Ning Mi, Lei Zhao

**Affiliations:** 1 School of Materials Engineering, Longdong University, Qingyang 745000, China; yangld777@126.com (L.Y.); ldxymining@126.com (N.M.); 2 Experimental Teaching Center of Mechanical Engineering, School of Intelligent Manufacturing, Longdong University, Qingyang 745000, China; niuxiaoqin1978@163.com

**Keywords:** supercapacitors, metal–organic framework, hollow nano-cage, nitrogen-doped carbon, electrode materials

## Abstract

Supercapacitors (SCs) have garnered significant attention due to their high power density and long cycle life. Among the various electrode materials, carbon materials have emerged as a focal point of research owing to their superior conductivity, stability, and reproducibility. However, the relatively low specific capacitance and specific surface area of carbon materials result in suboptimal electrochemical performance, which seriously hinders their practical applications. This work introduces a straightforward yet effective strategy for constructing hollow nano-cage structures by tannic acid etching of ZIF-8. In this process, tannic acid releases protons that selectively etch the MOF structure, while the residual large molecules adhere to the ZIF-8 surface, stabilizing its framework and preventing structural collapse. Following high-temperature heat treatment, novel hollow nitrogen-doped carbon nano-cage structures (HNCs) are successfully synthesized. Electrochemical tests reveal that the material has a capacity of 349.3 F g^−1^ at a current density of 0.5 A g^−1^, and still has a coulombic efficiency of 97.61% as well as a capacity retention of 97.86% after cycling for 10,000 cycles at a current density of 3 A g^−1^. Therefore, this study provides a novel way to explore the application of carbon materials with excellent electrochemical performance for energy storage.

## 1. Introduction

With the development of the global economy, the traditional industrial structure has undergone an obvious transformation, and electronic products [1,2] can be found everywhere in people’s everyday lives. In particular, the rapid development of the new energy industry has led more people to pay attention to energy storage devices; therefore, it is of strategic significance to seek clean [3], safe [4], and efficient energy storage technology [5]. As a novel energy storage device, supercapacitors address the limitations of low energy density for capacitors and of low power density for secondary batteries. These devices have garnered significant attention due to their high power density, long cycle life, and rapid charging and discharging capabilities. In supercapacitors, they can be classified into two categories based on distinct charge storage mechanisms: double-layer capacitors [6,7,8] and pseudocapacitors [9,10,11]. Double-layer capacitors store energy via the electrochemical adsorption/desorption of electrostatic charges at the interface between the electrode and the electrolyte. In contrast, pseudocapacitance refers to the occurrence of reversible redox reactions on the surface and near the surface of the active electrode material to generate Faraday quasi-capacitance for the storage of energy. Therefore, the choice of electrode materials is crucial for supercapacitors, which can directly affect the use of the device.

Carbon materials [12,13,14,15] have long been the most commonly used electrode materials for secondary batteries [16,17,18] and supercapacitors [19], with their advantages of high specific surface area, low cost, good electrical conductivity, and good electrochemical stability. However, the specific capacitance provided by carbon materials is very limited, and current studies have shown that structure modulation and heteroatom doping can significantly improve the electrochemical properties of carbon materials. Structure modulation primarily involves dimensional modulation, such as 0D materials [20,21], 1D materials [22,23], 2D materials [24,25], and 3D materials [24,26]. Zero-dimensional materials, which are mainly composed of quantum dots and carbon quantum dots, exhibit advantages such as small size and a larger contact area with the electrolyte. However, they suffer from lower conductivity and higher electrical resistance. One-dimensional materials, including carbon nanotubes and nanowires, possess the benefits of having lightweight properties and excellent mechanical stability, yet their conductivity is relatively low, and their chemical stability is insufficient. Two-dimensional materials, such as graphene and MXene, demonstrate superior electronic conductivity within the two-dimensional plane, high specific surface area, and structural stability. Nevertheless, they are prone to severe self-stacking, leading to a slow ion transport rate. Three-dimensional materials mainly include structures such as nanoflowers and metal–organic frameworks, which have the advantages of high specific surface area, low synthesis cost, easy functionalization, and other advantages due to which the high specific surface area can effectively provide more active sites, this being more conducive to the energy storage and conversion of electrode materials. Heteroatom doping [27,28,29] is mainly introduced through in situ carbonization, using high-temperature pyrolysis of nitrogen-containing precursors to introduce heteroatoms; this method is simple to operate and easy to control, and it can effectively regulate the specific capacity of carbon materials.

Metal–organic frameworks [30,31,32] (MOFs) and their derivatives serve as ideal precursors for fabricating electrode materials for high-performance supercapacitors, owing to their unique characteristics of high specific surface area and tunable pore structure. MOFs are a class of crystalline materials consisting of metal ions or metal clusters linked by organic ligands via coordination bonds, forming regular porous structures. The materials not only have high porosity and a tunable chemical composition but can also be transformed into porous carbon materials with high electrical conductivity by heat treatment. This transformation not only preserves the porous structure of MOFs but also introduces highly active electrochemical active sites, which provides a new idea for the design of supercapacitor electrode materials. Among them, ZIF-8 is composed of imidazole ligands and metal ions. Its structure features a high nitrogen content, large porosity, and excellent stability, making it an ideal precursor for the preparation of supercapacitor electrode materials. However, the direct high-temperature carbonization of ZIF-8 results in electrode materials with limited electrochemical performance and specific surface area, which significantly hinders ion transport and transfer in the electrolyte. Therefore, the electrochemical properties of ZIF-8 can be effectively optimized through structural modification. Wei et al. [33] prepared complex nickel–cobalt–manganese sulfide yolk–shell hollow spheres via a simple self-templating strategy. These exhibit a high specific capacitance of 1360 F g^−1^ at 1 A g^−1^ and superior rate capability when utilized as electrode materials for electrochemical supercapacitors. Long et al. [34] developed lignin@ZIF-8-based carbon with a hierarchical porous “core-shell” graphitized superstructure (KGC-EHL@ZIF-8) as an electrode material, achieving a maximum specific capacitance of 462.6 F g^−1^ at 0.5 A g^−1^ in a three-electrode system. Zhang et al. [35] employed nitrogen–sulfur co-doped ZIF-8 derivatives (NS-PCs) as the base material to fabricate a high-performance, low-self-discharge supercapacitor. Notably, the symmetric NS-PC-900//NS-PC-900 achieved a maximum specific capacitance of 42.7 F g^−1^. As a result, the selection of special materials as templates or precursors can effectively improve the electrochemical properties of electrode materials.

Hollow structures have received much attention in the structural modulation of many electrode materials. On the one hand, hollow structures can increase the specific surface area of the material and optimize ion transport; on the other hand, hollow structures can endow the electrode material with more active sites due to the larger volume per unit mass. Du et al. [36] prepared hollow carbon spheres using a simple hard template method, and simultaneously adjusted the surface chemical characteristics, surface roughness, and shell thickness, etc. In this process, the prepared electrode material had a capacity of 285 F g^−1^ at a current density of 0.5 A g^−1^. Tsai et al. [37] prepared hollow porous carbon microspheres (SPC-MnO_2_) by using sucrose as a carbon source and MnO_2_ microspheres as templates, which are activated by KHCO_3_. The SPC-MnO_2_ samples showed a large surface area of 1589 m^2^ g^−1^ and a pore volume of 2.52 cm^3^ g^−1^ and the resulting microporous mesoporous structure provided a hydrophilic surface, which enhanced wettability in the aqueous KOH electrolyte and improved the charge storage capacity. These exhibit a capacity of 317 F g^−1^ at a current density of 1 A g^−1^. Thus, the generation of hollow structures can significantly enhance the specific surface area of materials and thus improve the micro mesopores of electrode materials; these features can effectively facilitate ion/electron transport and surface-controlled redox kinetics. In the existing work, the construction of hollow structures is more complicated, with the hard template method, soft template method, sacrificial template method and template-free method usually being used; although these preparation processes are more mature, the results are uncontrollable and the materials are more expensive, which seriously limits the development of electrode materials. Tannic acid, as the most common weak acid in nature, is more stable for structural etching, and is non-toxic and harmless to the environment, so it has wider applicability.

In this work, novel hollow nitrogen-doped carbon nano-cage structures (HNCs) are prepared via the high-temperature pyrolysis of a composite precursor. ZIF-8 is employed as the framework precursor, while tannic acid serves as both an etchant and a nitrogen source. The hollow nano-cage structures (ZIF-8-TA) are obtained by compositing ZIF-8 with tannic acid at room temperature. During this process, protons released from tannic acid etch the MOF structure, while large molecules in tannic acid effectively stabilize the hollow nano-cage structure, preventing its collapse. The resulting etched hollow nano-cages exhibit a robust structural framework and rapid ion transport capability. They demonstrate a specific capacity of 349.3 F g^−1^ at a current density of 0.5 A g^−1^, a Coulombic efficiency of 97.61%, and a capacity retention of 97.86% after 10,000 cycles at a current density of 3 A g^−1^. Therefore, the electrochemical properties of carbon materials can be effectively improved by structural modulation and heteroatom doping, which can further broaden their application prospects.

## 2. Results and Discussion

**Figure 1:**
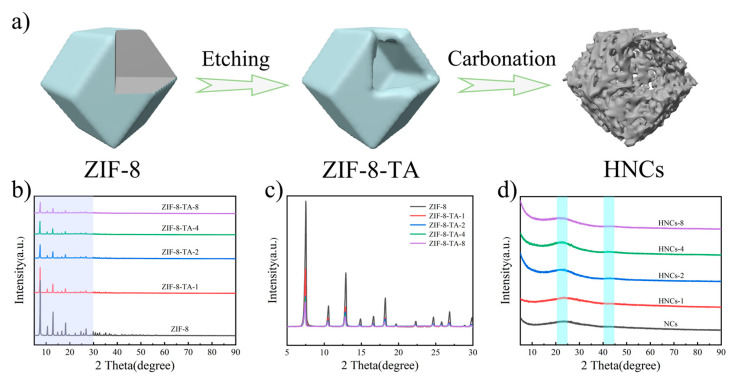
(**a**) Schematic diagram of composite material preparation of HNCs. (**b**) XRD patterns of the precursor before etching and after etching with different concentrations of tannic acid. (**c**) Partial diffraction magnification image of ZIF-8, ZIF-8-TA-1, ZIF-8-TA-2, ZIF-8-TA-4 and ZIF-8-TA-8. (**d**) XRD patterns after carbonation of NCs, HNCs-1, HNCs-2, HNCs-4, and HNCs-8.

Figure 1a presents a schematic illustration of the synthesis of HNCs. Firstly, the ZIF-8 precursor is prepared by the solvothermal method, followed by being etched using tannic acid. The tannic acid is exploited to release free protons that can be used to etch the MOF framework. Its relatively large molecular size allows it to block the pores of the MOF structure and ensure that the structure does not collapse completely. Also due to its larger molecular size, the tannic acid attached to the MOF surface can change the MOF surface from being hydrophobic to being hydrophilic, greatly improving the practical value of the MOF material. Then, the ZIF-8 material is etched by choosing different concentrations of tannic acid so as to achieve the purpose of constructing a special structure. Via pyrolysis of the etched material, ZIF-8-TA, at a high temperature, the hollow nitrogen-doped carbon nano-cage structure is finally obtained, and this can be applied as an electrode material in supercapacitors.

Figure 1b shows the XRD spectra [38] of ZIF-8 and samples etched with different concentrations of tannic acid. It is evident that tannic acid etching significantly reduces the intensity of the diffraction peaks of the ZIF-8 precursor, indicating that the etching process does not alter the original composition or structure of ZIF-8. Figure 1c shows an enlarged spectrum of the XRD from 5° to 30°, clearly demonstrating that as the concentration of tannic acid increases, the diffraction peaks of the ZIF-8 precursor become progressively weaker. Figure 1d shows the XRD spectrum after carbonation, in which obvious broad peaks appear at 23° and 43°, which are mainly attributed to the formation of amorphous carbon and prove that the composites were successfully prepared. In addition, the local structural changes in and defects of the different samples are further demonstrated by the Raman spectroscopy results provided in Figure S1a. Among others, the D peak is related to the lattice defects or disordered structure of the carbon material, while the G peak is related to the degree of graphitization of the carbon material. Therefore, the intensity of the ratio of the D peak to the G peak (I_D_/I_G_ ration) can be used to reveal the degree of defects in different electrode materials. The increase in the I_D_/I_G_ ratio with the increase in the degree of etching indicates that the introduction of tannic acid is favorable for increasing the pore structure of electrode materials, in turn leading to more active sites and thus improving the electrochemical performance of electrode materials. The specific surface area and pore size distribution of the materials are obtained using the nitrogen adsorption–desorption test method. According to Figure S1b, comparing the isothermal adsorption–desorption curves of the two materials, NCs and HNCs-8 belong to the type-IV and type-I isotherms, respectively, and after being etched with tannic acid, the specific surface area of NCs is increased from 543.19 m^2^ g^−1^ to 1, 133.59 m^2^ g^−1^. Moreover, it can be observed in the pore size distribution curves that micropores and mesopores existed in both materials (Figure S1c), with the size of the micropores being concentrated in the range of 0.3–2 nm. This further confirms the feasibility of etching precursors with tannic acid, a strategy that can effectively increase the specific surface area of NCs and enhance the microporous occupancy, thereby improving the electrochemical performance of electrode materials.

**Figure 2:**
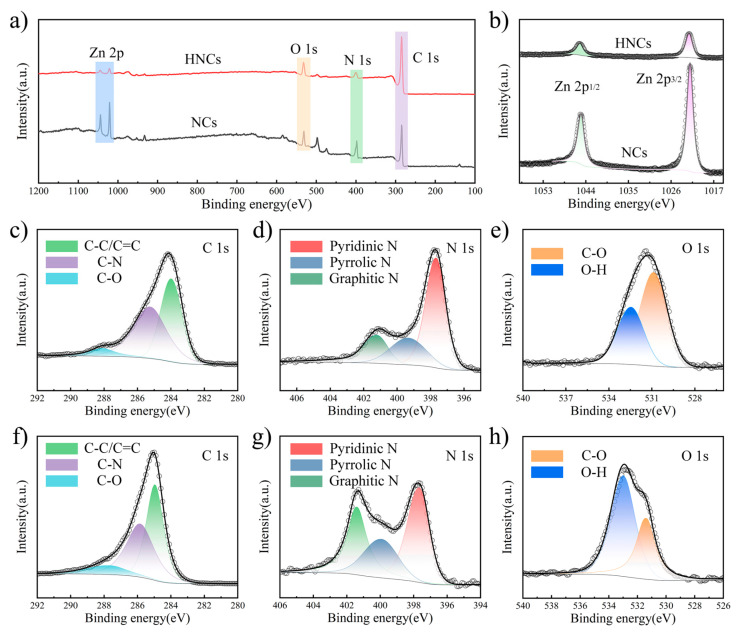
(**a**) XPS total spectrum of NCs and HNCs-8. XPS high-resolution spectra of (**b**) Zn 2p, (**c**) C 1s, (**d**) N 1s, and (**e**) O 1s of NCs. XPS high-resolution spectra of (**b**) Zn 2p, (**f**) C 1s, (**g**) N 1s, and (**h**) O 1s of HNCs.

X-ray photoelectron spectroscopy (XPS) is employed to analyze the elemental composition and ratios within the materials. As illustrated in Figure 2a, the XPS spectra of NCs and HNCs reveal the presence of four elements: C, N, O, and Zn. Among these, carbon exhibits the highest concentration, primarily due to the conversion of organic ligands in the precursors into carbon-based materials under high-temperature conditions. The specific ratios of these elements are detailed in Table S1. Figure 2b presents the detailed spectra of high-resolution NCs and HNCs. The high-resolution XPS spectra of Zn 2p can be separated into the following bonds: 1045.3 eV (Zn 2p^1/2^) and 1022.2 eV (Zn 2p^3/2^) [38]. By comparing the NCs and HNCs, it is evident that the intensity of the Zn element’s peaks is significantly reduced after etching. This indicates that tannic acid can remove a portion of the zinc ions, thus increasing the volume fraction per unit mass and laying a certain foundation for the excellent electrochemical performance. Figure 2c–e presents the detailed high-resolution spectra of NCs. The C 1s high-resolution XPS spectra can be separated into distinct peaks corresponding to the following bonds: 285 eV (C-C/C=C), 285.9 eV (C-N), and 287.8 eV (C-O). For the N 1s high-resolution XPS spectra, the deconvolution reveals the presence of bonds at 397.7 eV (pyridinic nitrogen), 400 eV (pyrrolic nitrogen), and 401.4 eV (graphitic nitrogen) [39]. Likewise, Figure 2f–h illustrate the fine spectra of HNCs. By analyzing and comparing the fine spectra of C 1s, N 1s, and O 1s, the presence of fitted C-N, C-O, O-H, and C=C bonds confirms the successful synthesis of the ZIF-8 precursor and the etched material. The analysis of the elemental content reveals that there is a significant decrease in the content of zinc after etching, corresponding to an increase in the content of carbon, which is in agreement with the conclusions obtained from XRD.

**Figure 3:**
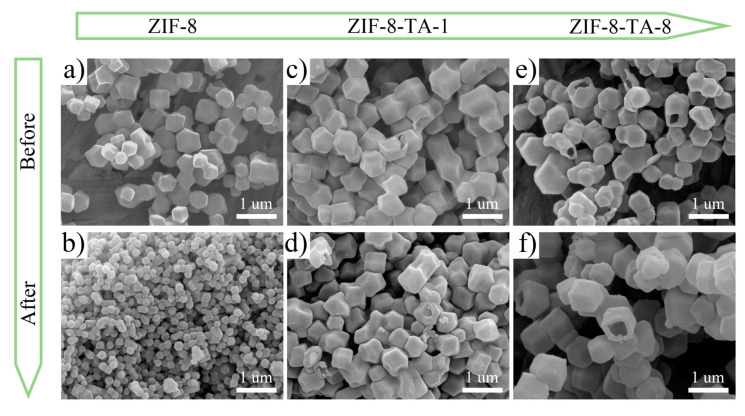
(**a**,**c**,**e**) SEM images of ZIF-8, ZIF-8-TA-1, and ZIF-8-TA-8 before carbonation. (**b**,**d**,**f**) SEM images of ZIF-8, ZIF-8-TA-1, and ZIF-8-TA-8 after carbonation.

To further investigate the effect of structure on the properties, Figure 3a–f present field emission electron microscopy images of ZIF-8, ZIF-8-TA-1, and ZIF-8-TA-8. Figure 3a,b show the scanning electron micrographs of ZIF-8 before and after carbonization. It is evident that the prepared precursor has a uniform dodecahedral structure. Furthermore, the individual structures are preserved within the nanostructures, with no formation of hollow structures observed either before or after carbonization. This confirms the successful preparation of the precursor ZIF-8. Figure S3 shows the EDS mapping analysis of the precursor ZIF-8 after carbonization, and it can be found that the four elements, C, O, N, and Zn, are uniformly distributed, with the contents being as follows: C (89.96%), N (1.06%), O (8.34%), and Zn (0.64%) (Table S2). Figure 3c,d display the scanning electron micrographs of ZIF-8-TA-1 before and after carbonization. These images reveal that the etching of the ZIF-8 precursor by a small dose of tannic acid is not obvious. However, a portion of the inner part of the structure of ZIF-8-TA-1 is etched away with respect to the precursor, which may also demonstrate that the tannic acid etching occurred from the outside to the inside, further exhibiting the controllable nature of the hollow nano-cage structure. Figure S2a–d show the scanning electron micrographs of ZIF-8-TA-2 and ZIF-8-TA-4 before and after carbonization, and it can be observed that the etching effect is more obvious with the increase in the amount of tannic acid as an etchant. Figure 3e,f show the scanning electron micrographs of ZIF-8-TA-8 before and after carbonization. It is evident that a hollow nano-cage structure forms when the concentration of tannic acid reaches 8 mg mL^−1^, thereby showing the feasibility of this strategy. Figure S4 shows the EDS mapping analysis of HNCs-8 with C (97.98%), N (1.03%), and O (0.99%) (Table S2), which is in agreement with the XPS test results in Figure 2. Given that the hollow structure is composed of inner and outer layers, this structure can give the material a larger specific surface area as well as active sites, which will be favorable for the adsorption and desorption of ions. Moreover, the nanosized structure can effectively shorten the ion transport path and effectively improve the efficiency of the electrode material.

**Figure 4:**
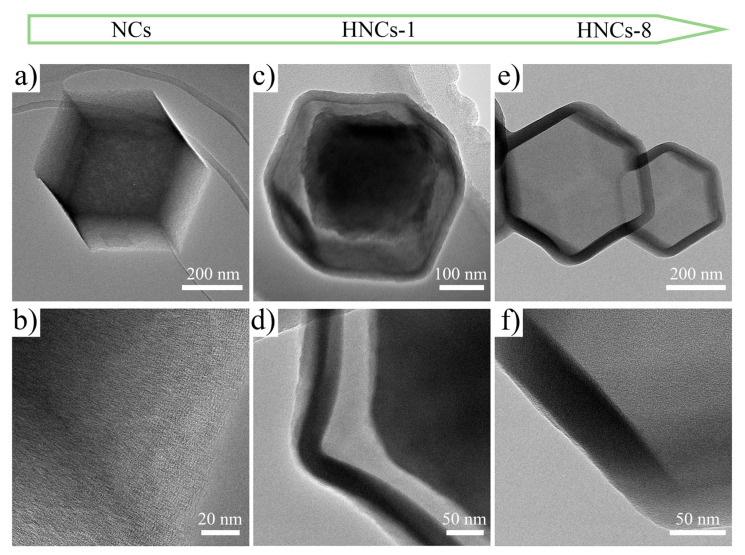
(**a**–**f**) TEM image of NCs, HNCs-1 and HNCs-8 at different magnifications.

Figure 4a–f show the transmission electron microscope (TEM) images of ZIF-8, ZIF-8-TA-1, and ZIF-8-TA-8 after carbonization, which are named NCs, HNCs-1, and HNCs-8, respectively. Figure 4a,b show the TEM pictures of the ZIF-8 precursor after carbonation. Due to the chelation between metal ions and organic ligands forming ZIF-8 nanoparticles, it can be observed that the precursor after carbonization has a morphology consistent with that observed in scanning electron microscopy; it is entirely dodecahedral in structure, which indicates that the preparation of the precursor for this material was successful. A high density of pores on the surface of the carbonization NCs can be observed in the HRTEM of Figure 4b, and this porous structure offers promising potential for the development of high-capacity electrode materials. With the introduction of tannic acid as an etchant, the dodecahedral structure of HNCs-1 in Figure 4c can be observed to gradually undergo etching. Compared to the dodecahedral structure of NCs, HNCs-1 gradually transitions into having a hollow structure to produce a core–shell structure. It is due to the release of free protons by tannic acid that the effect of etching is produced on the structure of the NCs; the larger molecular size of the tannic acid effectively prevents the destruction of the shells, and the core–shell structure of the HNCs-1 material can be further observed in the HRTEM images in Figure 4d, proving the feasibility of this strategy. As the concentration of tannic acid increased, the etching effect became more significant. Figure S5a,b show the TEM images of HNCs-2. In Figure S5a, it can be observed that the core–shell structure produced by etching changed significantly compared with that of HNCs-1, and the nucleus structure of HNCs-2 gradually became smaller. Figure S5b shows the HRTEM images, which make it evident that HNCs-2 exhibits a more abundant pore distribution on its surface. This feature allows it to effectively improve the reaction kinetics of electrode materials. Figure S5c,d show the TEM images of HNCs-4, revealing a further reduction in the nuclear structure, which validates the feasibility of the precursor strategy of etching with tannic acid. Figure 4e,f display the TEM pictures of HNCs-8, where it can be found that, with a further increase in etchant content, the nucleus structure disappeared, generating a hollow hallow nano-cage structure with a larger specific surface area relative to that of the NCs and e more active sites, allowing it to effectively shorten ionic transport paths and optimize interfacial reaction kinetics.

**Figure 5:**
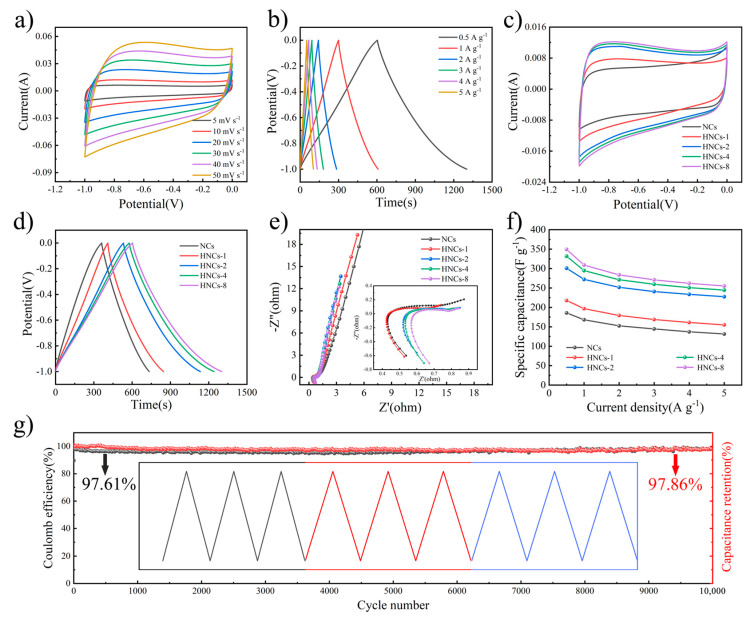
(**a**) CV curves of HNCs-8 at various scan rates from 5 to 50 mV s^−1^. (**b**) GCD curves at different current densities from 0.5 to 5 A g^−1^ of HNCs-8. (**c**) A CV comparison diagram of different materials at different current densities. (**d**) A GCD comparison diagram of different materials at different current densities. (**e**) EIS comparison of different materials. (**f**) Comparison of specific capacity of different materials at different current densities. (**g**) Coulomb efficiency and capacity retention change diagram after 10,000 cycles.

In order to verify the potential of HNCs for electrochemical applications, the electrochemical properties of HNCs as electrode materials are explored. NC and HNC samples with varying compositions are employed as working electrodes for single-electrode testing. These electrodes undergo characterization through cyclic voltammetry (CV), constant current charging and discharging (GCD) curves, and electrochemical impedance spectroscopy (EIS) to assess their performance [40]. Figure S6a shows the CV curves of the NCs at scan rates ranging from 5 to 50 mV s^−1^, and the obvious rectangle-like characteristics of the CV curves can be observed, without obvious redox peaks. Figure S6b, showing the results of the GCD test, reveals that the curves exhibit excellent symmetry, so this also proves that NCs are more reversible and exhibit good double-layer capacitance characteristics. The discharging process is carried out at current densities ranging from 0.5 to 5 A g^−1^. The capacities of the NCs are calculated using Equation (1), yielding values of 185.8 F g^−1^, 168.1 F g^−1^, 152.6 F g^−1^, 144.6 F g^−1^, 137.2 F g^−1^, and 131.5 F g^−1^. After carbonization, ZIF-8 maintains its dodecahedral morphology along with its porous architecture, which enhances the electrochemical performance of the NCs. The CV and GCD curves for HNCs-1, HNCs-2, and HNCs-4 are presented in Figure S6c–f, and Figure S7a,b, respectively. These figures indicate that the electrochemical characteristics of HNCs-1, HNCs-2, and HNCs-4 vary significantly with alterations in the quantity of tannic acid. Figure 5a,b shows the CV and GCD test curves of HNCs-8; a rectangular shape can be observed in the CV curve in Figure 5a, indicating that the energy storage mechanism of this material is consistent with that of the NC material, which is attributed to the capacitive behavior of the double layer. Figure 5b presents the GCD curves of HNCs-8 under different current densities, demonstrating its excellent reversibility. The specific capacities of HNCs-8 are calculated using Equation (1), and these are found to be 349.3 F g^−1^, 308.9 F g^−1^, 283.8 F g^−1^, 270.9 F g^−1^, 262.0 F g^−1^, and 255.0 F g^−1^. Compared with the NCs precursor electrode material, the specific capacity of HNCs-8 is enhanced by nearly two times at a current density of 0.5 A g^−1^, which validates the feasibility of preparing hollow nano-cage structures by tannic acid etching. Figure 5c illustrates a comparison of the CV curves for different tannic acid contents, through which it can be found that the electrochemical properties exhibited by different electrode materials are consistent. Figure 5d shows a comparison of the GCD curves with different tannic acid contents, and it can be found that the area of HNCs-8 is the largest, and the discharge time is the longest; therefore, the electrochemical performance of HNCs-8 is also superior. Subsequently, EIS is carried out, as shown in Figure 5e, and it can be found that the slope of these kinds of materials is larger in the low-frequency region, demonstrating the excellent ion diffusion rate of these carbon materials. This also shows that the diffusion impedance of HNCs-8 electrode materials is smaller compared to that of NC electrode materials, which suggests that hollow nano-structures can effectively increase porosity as well as shorten the ion diffusion path. In the high-frequency region, the radius of the semicircular region (charge transfer impedance *Rct*) and the intercept with the x-axis (intrinsic impedance *Rs*) in the spectra are very small, which indicate excellent interfacial reaction kinetics. Figure S8 and Table S3 are the EIS results combined with the equivalent circuit fitting results. Figure 5f and Table S4 show the plots of the rate performance tests on different samples at different current densities, and the electrochemical performance of this work is excellent compared to that in the reported work (Table S5) [41,42,43,44,45,46]. Figure 5g shows the long-cycle test conducted on HNCs-8, and it can be seen that HNCs-8 still has relatively stable Coulombic efficiency and capacity retention after cycling at a current density of 3 A g^−1^ for 10,000 cycles. Therefore, the electrochemical test reveals that the electrochemical performance of HNCs-8 is significantly enhanced compared to that of carbon NCs, further demonstrating the potential of hollow nano-spheres for long-life energy storage devices.

## 3. Experiment

### 3.1. Materials and Chemicals

Zinc nitrate hexahydrate (99.0%, Zn(NO_3_)_2_·6H_2_O) was purchased from Tianjin Damao Chemical Reagent Factory. Tannic acid and dimethylimidazole (98%) were purchased from Shanghai Maclean Biochemical Technology Co., Ltd., Shanghai, China, Methanol was obtained from Tianjin Best Chemical Co., Ltd., Tianjin, China. All reagents used in the experiments were of analytical grade, and the water used was homemade laboratory-deionized water.

### 3.2. Synthesis of Materials

Synthesis of ZIF-8 precursor: ZIF-8 was synthesized by the traditional solvothermal method. Firstly, 1.116 g of zinc nitrate hexahydrate was dissolved in 30 mL of methanol solution, which was recorded as solution A. Separately, 1.232 g of dimethylimidazole was dissolved in 30 mL of methanol solution to prepare solution B. Subsequently, solutions A and B were homogeneously mixed and ultrasonically dispersed for 6 min to ensure complete dissolution and uniform distribution. The resulting mixture (solution AB) was then transferred to a 100 mL stainless steel autoclave lined with PTFE. The sealed autoclave was heated at 120 °C for 2 h in an oven. After cooling to room temperature, the product was collected by centrifugation, washed sequentially with methanol and deionized water, and finally dried in a vacuum oven at 60 °C. The final product obtained was ZIF-8.

Synthesis of ZIF-8-TA: We took 0.1 g of the ZIF-8 material mentioned above and put it in 1 g L^−1^ tannic acid (TA) solution. After aging for 10 min, the product was washed with methanol and deionized water, and put into a 60 °C vacuum oven to dry. Finally, the product, ZIF-8-TA, was obtained.

Synthesis of NCs: The dried ZIF-8 was subjected to pyrolysis at 800 °C for 2 h in a nitrogen atmosphere to finally obtain NCs.

Synthesis of HNCs: The dried ZIF-8-TA-X was subjected to pyrolysis at 800 °C for 2 h under a nitrogen atmosphere, finally resulting in the production of HNCs. The numbers indicate varying concentrations of tannic acid (for example, number 8 corresponds to a tannic acid concentration of 8 g L^−1^).

### 3.3. Characterization of Materials

The structural characteristics were analyzed using transmission electron microscopy (TEM, JEM-F200, JEOL, Tokyo, Japan) and scanning electron microscopy (SEM, JSM-6701F, JEOL, Tokyo, Japan), as is typically carried out. Powder X-ray diffraction (XRD) data were collected with a D/MAX-2400 diffractometer (Rigaku, Tokyo, Japan) within the range of 5–90°, employing a step size of 0.02 and a scanning speed of 10°/min. X-ray photoelectron spectroscopy (XPS) measurements were performed using a VG ESCALAB 250 Xi spectrometer (Thermo Fisher Scientific Inc., Waltham, MA, USA). The Raman spectra were recorded by a LabRAM HR Evolution Raman spectrophotometer (HORIBA Jobin Yvon S.A.S., Palaiseau, France). The specific surface area and pore size distribution of different samples were assessed using the Brunauer–Emmett–Teller (BET) and Saito–Foley (SF) approaches, respectively.

### 3.4. Electrochemical Measurements

Single Electrode Testing: The preparation of the working electrode involved a mass ratio of 80% active electrode material, 7.5% acetylene black, 7.5% graphite, and 5% polytetrafluoroethylene (PTFE). These components were mixed and stirred to form a uniform paste. During the mixing process, a small amount of alcohol was added to aid dispersion. The resulting paste was then coated onto a 1 cm² area of nickel foam substrate to fabricate the working electrodes, with each electrode containing 4 mg of active material. Following this, the electrodes were dried under vacuum at 60 °C for 8 h and subsequently pressed at 10 MPa for 30 s to enhance the contact between the active material and the current collector. The electrochemical properties of the electrodes were evaluated at room temperature using a CHI660E electrochemical workstation. The testing setup included a 6 M potassium hydroxide electrolyte and a platinum sheet as the counter electrode in a single-electrode configuration. Cyclic voltammetry measurements were performed at various scan rates ranging from 5 to 50 mV s^−1^. Additionally, constant current charging and discharging tests were conducted at current densities varying from 0.5 to 5 A g^−1^. Electrochemical impedance spectroscopy (EIS) analysis was carried out within a frequency range of 10^−2^–10^5^ Hz. For cyclic stability assessment, the LAND CT2001A system was employed, and the specific capacitance was calculated using Equation (1) most of the time:

(1) $$C=\frac{I\;\times \;∆t}{m\;\times \;∆V}$$

where *C* (F g^−1^) is the specific capacitance, *I* (A) is the current, *m* (mg) is the mass of the active substance, Δ*V* (V) was the voltage window, and Δ*t* (s) is the discharging time.

## 4. Conclusions

In summary, this work successfully demonstrates the feasibility of constructing hollow nano-cage structures by etching ZIF-8 with tannic acid. In this process, tannic acid releases protons to etch the MOF structure while its relatively large molecules adsorb onto the surface of ZIF-8, thereby stabilizing its structure and preventing collapse. After high-temperature heat treatment, novel hollow nitrogen-doped carbon nano-cage structures (HNCs) are obtained. This structure has a capacity of 349.3 F g^−1^ at a current density of 0.5 A g^−1^ and still has a Coulombic efficiency of 97.61% and a capacity retention of 97.86% after cycling 10,000 cycles at a current density of 3 A g^−1^. Thus, the electrochemical properties of carbon materials can be significantly enhanced through structural modulation and heteroatom doping. This innovative structure offers insights into the development of next-generation electrode materials, showcasing extensive application potential.

## Data Availability

The original contributions presented in this study are included in the article/supplementary material. Further inquiries can be directed to the corresponding author(s).

## Supplementary Materials

The following supporting information can be downloaded at: https://www.mdpi.com/article/10.3390/molecules30102130/s1, Figure S1: (a) Raman analysis of different materials, (b) Nitrogen adsorption-desorption isotherms, and (c) Pore size distribution curves of NCs and HNCs-8; Figure S2: (a,c) are SEM images of ZIF-8-TA-2 and ZIF-8-TA-4 before carbonation. (b,d) are SEM images of ZIF-8-TA-2 and ZIF-8-TA-4 after carbonation; Figure S3: SEM-EDS mapping analysis of NCs; Figure S4: SEM-EDS mapping analysis of HNCs-8; Figure S5: a-b) and c-d) At different multiples TEM image of HNCs-2 and HNCs-4; Figure S6: (a,c,e) At different scan rates CV curves of NCs, HNCs-1, and HNCs-2. (b,d,f) At different current densities GCD curves of NCs, HNCs-1, and HNCs-2; Figure S7: (a) At different scan rates CV curves of HNCs-4. (b) At different current densities GCD curves of HNCs-4; Figure S8: EIS impedance modelling of different materials; Table S1: Elemental percentage of the NCs and HNCs-8; Table S2: SEM-EDS mapping percentage of elemental content; Table S3: EIS combined with equivalent circuit fitting results; Table S4: Specific capacitance at different current densities; Table S5: Comparison of the specific capacitance of carbon materials.
