# Supplementary material for: Controllable Nitrogen-Doped Hollow Carbon Nano-Cage Structures as Supercapacitor Electrode Materials
Source: Molecules. 2025 May 12;30(10):2130. doi: 10.3390/molecules30102130 (PMC12114442; doi:10.3390/molecules30102130)
Supplement: Supplementary file 1 [file molecules-30-02130-s001.zip › molecules-3568142-supplementary.pdf]

Support Information

Controllable Nitrogen-Doped Hollow Carbon Nano-Cage Structures as Supercapacitor Electrode Materials

Yitong Sun*, Xiaoqin Niu, Laidong Yang, Ning Mi, Lei Zhao*

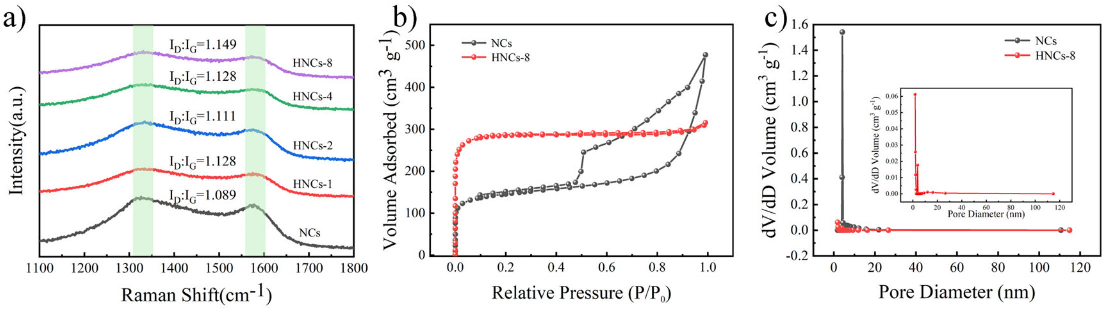

Figure S1. *a) Raman analysis of different materials, b) Nitrogen adsorption-desorption isotherms, and c) Pore size distribution curves of NCs and HNCs-8.*

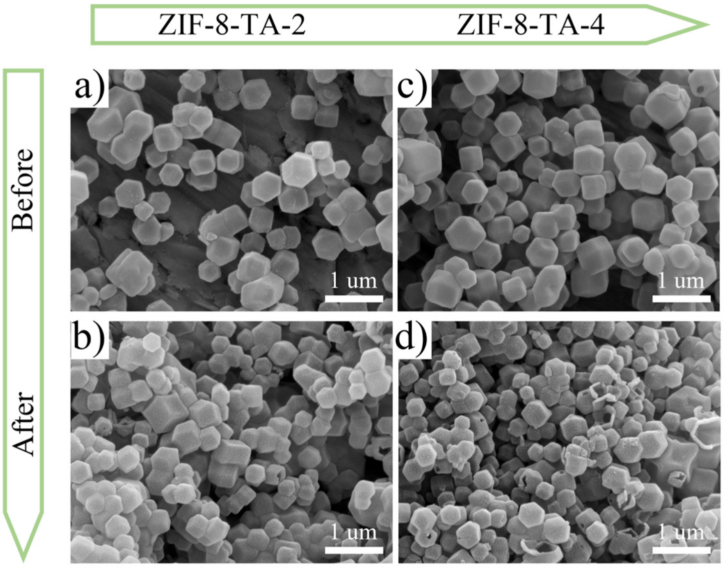

Figure S2. *a) and c) are SEM images of ZIF-8-TA-2 and ZIF-8-TA-4 before carbonation. b) and d) are SEM images of ZIF-8-TA-2 and ZIF-8-TA-4 after carbonation.*

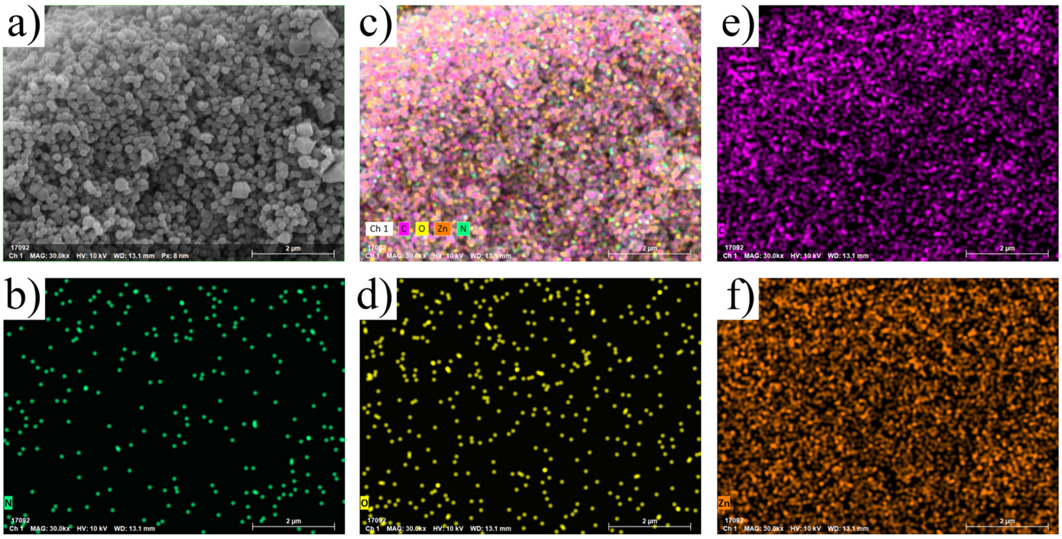

Figure S3. SEM-EDS mapping analysis of NCs.

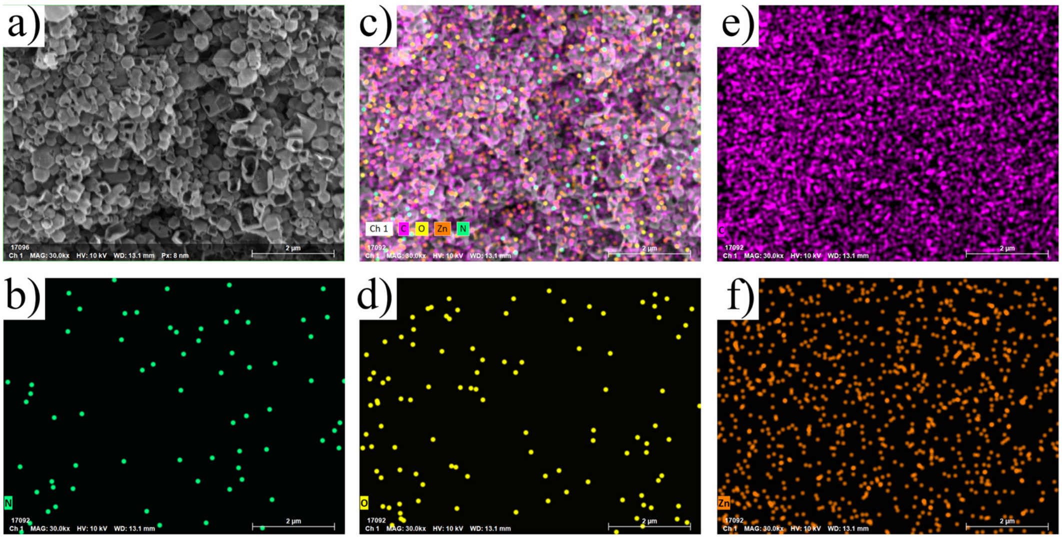

Figure S4. *SEM-EDS mapping analysis of HNCs-8.*

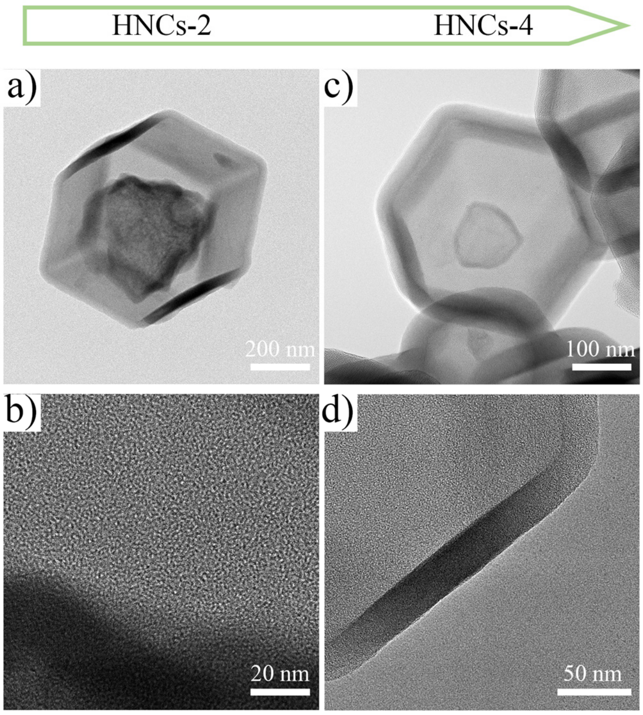

Figure S5. *a-b) and c-d) At different multiples TEM image of HNCs-2 and HNCs-4.*

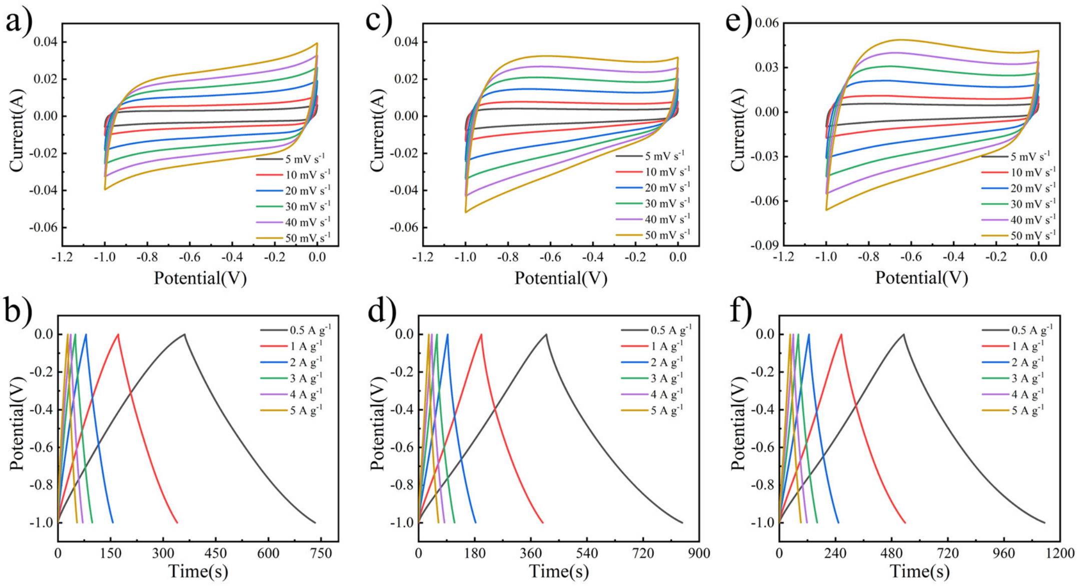

Figure S6. *a), c), and e) At different scan rates CV curves of NCs, HNCs-1, and HNCs-2. b), d), and f) At different current densities GCD curves of NCs, HNCs-1, and HNCs-2.*

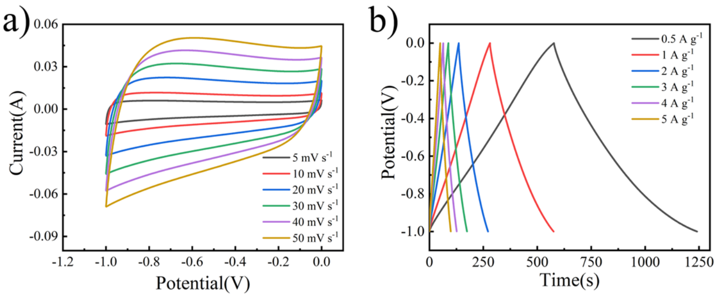

Figure S7. *a) At different scan rates CV curves of HNCs-4. b) At different current densities GCD curves of HNCs-4.*

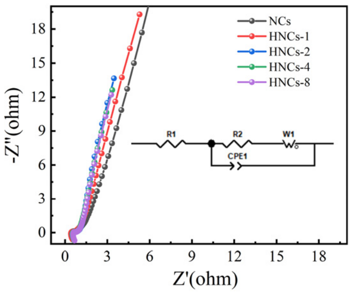

Figure S8. *EIS impedance modelling of different materials.*

Table S1. *Elemental percentage of the NCs and HNCs-8*

Materials \ Element Atom (%)	C 1s	N 1s	O 1s	Zn 2p
NCs	65.75	19.68	10.28	4.29
HNCs-8	86.57	5.03	7.99	0.40

Table S2. *SEM-EDS mapping percentage of elemental content.*

Materials \ Element Atom (%)	C	N	O	Zn
NCs	89.96	1.06	8.34	0.64
HNCs-8	97.98	1.03	0.99	0.00

Table S3. *EIS combined with equivalent circuit fitting results.*

Sample	R_s (Ω)	R_{ct} (Ω)	Z_w (Ω)
NCs	0.46	0.10	1.63
HNCs-1	0.46	0.09	1.18
HNCs-2	0.56	0.17	0.95
HNCs-4	0.57	0.13	0.78
HNCs-8	0.45	0.15	0.55

Table S4. *Specific capacitance at different current densities.*

Materials \ Current density	0.5 A g^{-1}	1 A g^{-1}	2 A g^{-1}	3 A g^{-1}	4 A g^{-1}	5 A g^{-1}
NCs	185.8 F g^{-1}	168.1 F g^{-1}	152.6 F g^{-1}	144.6 F g^{-1}	137.2 F g^{-1}	131.5 F g^{-1}
HNCs-1	217.8 F g^{-1}	196.7 F g^{-1}	179.2 F g^{-1}	168.9 F g^{-1}	161.2 F g^{-1}	155 F g^{-1}
HNCs-2	300.75 F g^{-1}	272 F g^{-1}	251.6 F g^{-1}	240.9 F g^{-1}	233.6 F g^{-1}	227.5 F g^{-1}
HNCs-4	331.5 F g^{-1}	294.6 F g^{-1}	271.2 F g^{-1}	259.5 F g^{-1}	250.8 F g^{-1}	244.5 F g^{-1}
HNCs-8	349.3 F g^{-1}	308.9 F g^{-1}	283.8 F g^{-1}	270.9 F g^{-1}	262.0 F g^{-1}	255.0 F g^{-1}

Table S5. *Comparison of the specific capacitance of carbon materials.*

Electrode material	Current density	Specific capacitance	Electrolyte	Reference
Nitrogen-Doped Carbon Nano-Hollow Cage	0.5 A g^{-1}	349.3 F g^{-1}	6 M KOH	This work
interconnected carbon nanocages	1 A g^{-1}	194 F g^{-1}	$EMIMBF_4$, 98%	[41]
Nitrogen-doped hollow carbon spheres	0.2 A g^{-1}	368 F g^{-1}	6 M KOH	[42]
nitrogen-doped hollow carbon spheres	0.5 A g^{-1}	213 F g^{-1}	6 M KOH	[43]
Nitrogen-doped hollow carbon sphere	1 A g^{-1}	170 F g^{-1}	6 M KOH	[44]
Hierarchical hollow microspheres of carbon nanorods	0.5 A g^{-1}	287 F g^{-1}	2 M H_2SO_4	[45]
Raspberry-like hollow carbon spheres	1 A g^{-1}	257 F g^{-1}	6 M KOH	[46]
